# Indocyanine Green Fluorescence Using in Conduit Reconstruction for Patients With Esophageal Cancer to Improve Short-Term Clinical Outcome: A Meta-Analysis

**DOI:** 10.3389/fonc.2022.847510

**Published:** 2022-06-01

**Authors:** Zhi-Nuan Hong, Liqin Huang, Weiguang Zhang, Mingqiang Kang

**Affiliations:** ^1^ Department of Thoracic Surgery, Fujian Medical University Union Hospital, Fuzhou, China; ^2^ Key Laboratory of Cardio-Thoracic Surgery (Fujian Medical University), Fujian Province University, Fuzhou, China; ^3^ Key Laboratory of Ministry of Education for Gastrointestinal Cancer, Fujian Medical University, Fuzhou, China; ^4^ Fujian Key Laboratory of Tumor Microbiology, Fujian Medical University, Fuzhou, China; ^5^ Department of Equipment, Fujian Medical University Union Hospital, Fuzhou, China

**Keywords:** esophagectomy, indocyanine green, anastomotic leak, meta-analysis, short-term outcome

## Abstract

**Objectives:**

This meta-analysis evaluated the short-term safety and efficacy of indocyanine green (ICG) fluorescence in gastric reconstruction to determine a suitable anastomotic position during esophagectomy.

**Methods:**

The Preferred Reporting Items for Systematic Reviews and Meta-Analyzes 2020 (PRISMA) were followed for this analysis.

**Results:**

A total of 9 publications including 1,162 patients were included. The operation time and intraoperative blood loss were comparable in the ICG and control groups. There was also no significant difference in overall postoperative mortality, reoperation, arrhythmia, vocal cord paralysis, pneumonia, and surgical wound infection. The ICG group had a 2.66-day reduction in postoperative stay. The overall anastomotic leak (AL) was 17.6% (n = 131) in the control group and 4.5% (n = 19) in the ICG group with a relative risk (RR) of 0.29 (95% CI 0.18–0.47). A subgroup analysis showed that the application of ICG in cervical anastomosis significantly reduced the incidence of AL (RR of 0.31, 95% CI 0.18–0.52), but for intrathoracic anastomosis, the RR 0.35 was not significant (95% CI 0.09–1.43). Compared to an RR of 0.35 in publications with a sample size of <50, a sample size of >50 had a lower RR of 0.24 (95% CI 0.12–0.48). Regarding intervention time of ICG, the application of ICG both before and after gastric construction had a better RR of 0.25 (95% CI 0.07–0.89).

**Conclusions:**

The application of ICG fluorescence could effectively reduce the incidence of AL and shorten the postoperative hospital stay for patients undergoing cervical anastomosis but was not effective for patients undergoing intrathoracic anastomosis. The application of ICG fluorescence before and after gastric management can better prevent AL.

**Systematic Review Registration:**

PROSPERO, CRD:42021244819.

## Introduction

Esophagectomy is an important means of radical/curative treatment of esophageal cancer. Among the postoperative complications, anastomotic leakage (AL) after esophagectomy remains a risk of considerable morbidity and mortality. In high-volume centers, AL rates range from 5 to 40%, even up to 50% in some medical centers, despite surgical advances and preoperative optimizations (1–4). Among the risk factors affecting anastomotic integrity, poor perfusion is a factor that can be intervened upon surgically. Gastric tube blood supply is currently monitored clinically by monitoring blood vessel color, temperature, and arterial pulse to predict poor perfusion. However, these parameters cannot reliably and objectively reflect the level of perfusion, therefore they have limited predictive value (5, 6).

Thus, it would be useful to find valid parameters to evaluate the perfusion status. Indocyanine green (ICG) is a water-soluble three-carbon anthocyanin dye with a plasma half-life span of 3–5 min. ICG absorbs light at an excitation wavelength of between 750 and 800 nm while emitting light at longer emission wavelengths of 830 nm or more. Only a few patients developed anaphylactic shock after an intravenous injection of ICG. Regarding its safety concerns, ICG has been added to the rapid food and drug administration approval for clinical use (7, 8). Nowadays, the application of ICG fluorescence angiography in the resection of esophageal carcinoma and conduit reconstruction is being widely developed.

At present, ICG fluorescence has been widely used in liver surgery, sentinel lymph node biopsy of breast cancer, and gastric cancer. In the field of thoracic surgery, it is mainly used in the location of pulmonary nodules, determination of pulmonary segment boundaries during pulmonary segmentectomy, sentinel lymph node location in the thoracic cavity of lung cancer, intraoperative chest guide display, and gastric perfusion assessment (9–12). Previous meta-analysis has confirmed that ICG fluorescence is an objective and useful parameter for evaluating gastric microcirculation (13, 14). This study aimed to systematically review the existing literature to determine the value of ICG fluorescence for short-term efficacy, especially for preventing anastomotic leakage (AL), and to investigate whether there are differences in the efficacy of ICG among different anastomosis sites (intrathoracic versus cervical anastomosis), the sample size in the intervention group (<50 versus >50), and the intervention time (only after tube construction versus both before and after tube construction).

## Method

A meta-analysis was conducted according to the Preferred Reporting Items for Systematic Reviews and Meta-Analysis statement (PRISMA) (15). IRB approval and written consent were not required for this further analysis.

### Literature Search Method

A search of PubMed, Embase, Web of Science, The Cochrane Library, Chinese National Knowledge Infrastructure, VIP Database, China Biology Medicine Disc, and Wan-fang database was conducted on 10 April 2021 by two independent researchers. The retrieval terms included ICG and esophagectomy. Both database subject heading fields (Emtree in EMBASE, MeSH in MEDLINE) and text word fields were searched. References of retrieved articles and reviews were also manually screened to obtain additional relevant studies.

### Study Selection

The inclusion criteria included: (1) esophagectomy with gastric conduit reconstruction; (2) comparative study design: control group using color, temperature, and pulsation of vessels; experimental group using ICG to assess perfusion; (3) age ≥18 years; (4) sufficient clinical outcome data for further analysis; and (5) sample size ≥10. The exclusion criteria were as follows: (1) abstract or review; (2) data insufficient; (3) repeated publications; and (4) included in a multicenter study.

### Quality Assessment

We used the Methodological Index for Non-Randomized Studies tool (MINORS) (score from 0 to 24). MINORS is a 12-point validated tool designed specifically to evaluate the methodological quality of non-randomized surgical trials. For the comparative studies, the ideal score was 24. A MINORS score below 12 indicates poor quality. A MINORS score above 18 indicates good quality (16, 17). Disagreements in the quality assessment were resolved through discussion.

### Data Extraction

Data were extracted by two independent researchers (Z-NH and LH) and entered into an EXCEL file including author, published time, country, study design, BMI, sample size, gender, history of smoking, American Society of Anesthesiologists (ASA) status, histological type, preoperative albumin, preoperative comorbidity (including hypertension, diabetes mellitus, obstructive lung disease), neoadjuvant therapy, tumor location, pathological tumor category, TNM stage, anastomotic method (stapler/hand sewn), route of gastric conduit, operation type, operative time, intraoperative blood loss, AL number, other complications, postoperative hospital stay, and hospital cost.

### Statistical Analysis

We used the inconsistency statistic (I^2^) to evaluate the extent of heterogeneity. Relative risk was used to evaluate the binary variable and the weighted mean difference for the continuous variable. An I^2^ value greater than 50% was considered to indicate substantial heterogeneity. A fixed model was used when I^2^ <50%, and a random model was used when I^2^ ≧50%. A 2-sided test at the 5% level was defined as indicating statistical significance. We only calculated the existing data and did not fill in the missing data. Subgroup and sensitivity analyses were conducted to find the heterogeneity source. Publication bias would be assessed by funnel plots, Begg’s test, and Egger’s tests. Statistical analysis was conducted with Stata version 15 (Stata Corp, College Station, TX, USA), and Revman 5.4.

## Results

### Study Selection

**Figure 1:**
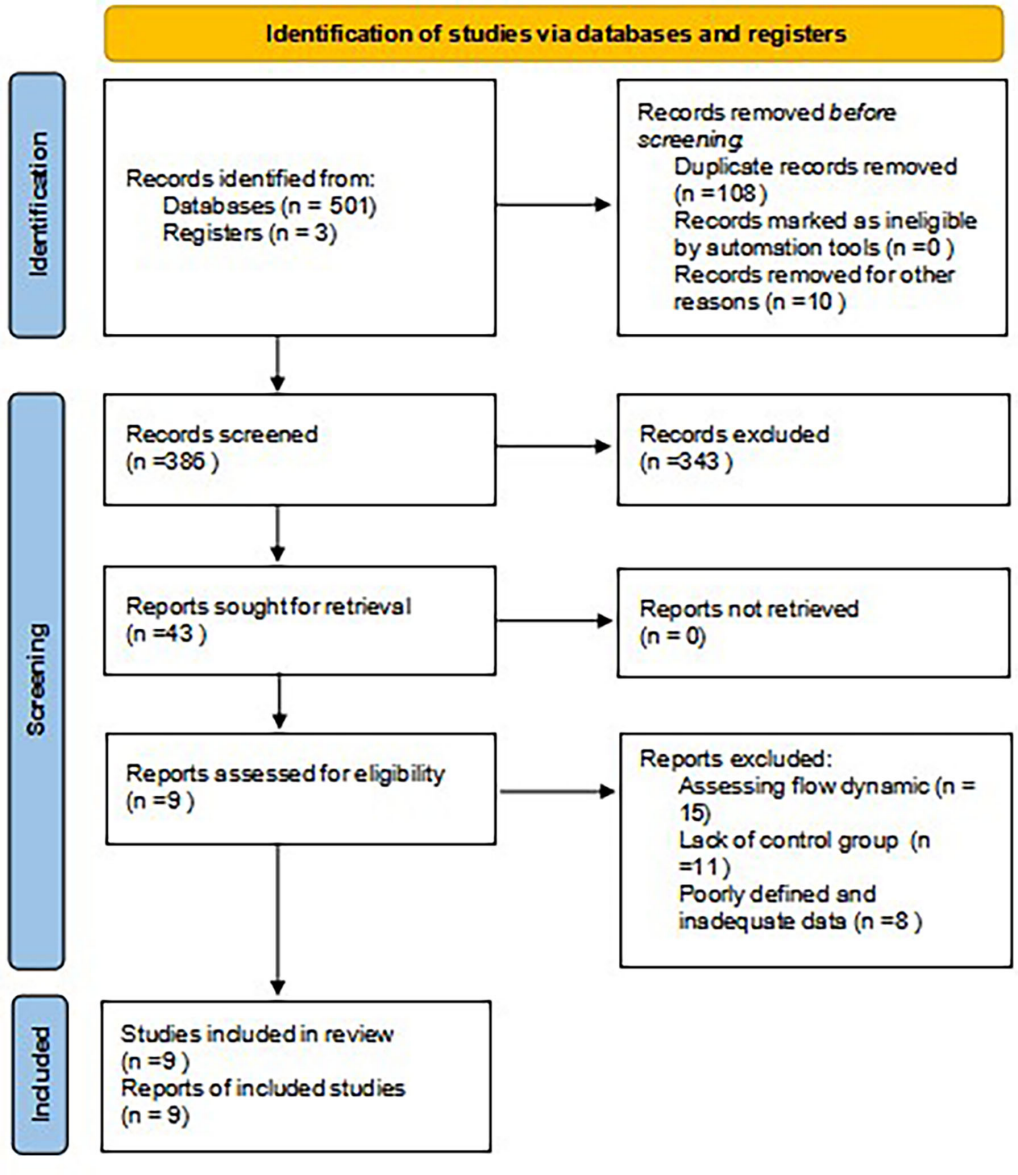
PRISMA flow diagram. PRISMA, Preferred Reporting Items for Systematic Reviews and Meta-Analysis.

Electronic database search results are available in the PRISMA flow diagram (**Figure 1**). From an initial total of 501 studies, 42 underwent full-text review, with 9 studies being included in our analysis (18–26). Many related publications focus on the flow dynamics of ICG perfusion.

### Study Characteristics

A total of 1,162 patients from 4 countries were included in our analysis, of whom 419 patients underwent ICG. Although most of the publications were retrospective, there was only 1 prospective study. The sample size ranged from 40 to 285. There was some heterogeneity regarding the ICG dose, near-infrared system, operation type, and ICG intervention time. The ICG and near-infrared system were varied, which makes it difficult for further analysis. Detailed information about the included studies can be found in **Table 1**.

**Table 1:** Baseline characteristics of included studies.

	Included studies	Heterogeneity		Model	Pooled effect	
		*p*	I2%		95% CI	*p*
Male	8	0.49	0	Fixed	0.93 (0.67-1.29)	0.49
Age	6	0.03	59.8	Random	0.07 (-0.08-0.22)	0.35
BMI	4	0.43	0	Fixed	0.12 (-0.08-0.31)	0.25
History of smoking	2	0.92	0	Fixed	1.12 (0.70-1381)	0.63
ASA I-II	3	0.1	51.7	Random	0.63 (0.4-0.99)	0.04
SSC	6	0.34	7.6	Fixed	0.98 (0.56-1.71)	0.94
Diabetes mellitus	3	0.86	0	Fixed	1.14 (0.78-1.66)	0.5
Cardiovascular disease	3	0.27	24	Fixed	1.08 (0.60-1.94)	0.79
Obstructive lung disease	3	0.63	0	Fixed	0.37 (0.13-1.03)	0.06
Neoadjuvant therapy	6	0.01	65	Random	0.59 (0.41-0.85)	0.004
Tumor in upper thoracic	3	0.07	62.6	Random	0.86 (0.51-1.47)	0.59
Pathological Tumor Category T1-2	3	0.13	51	Random	0.87 (0.57-1.32)	0.51
TNM stage I-II	4	0.006	75.6	Random	0.86 (0.60-1.23)	0.4
Thoracoscopy	6	0.72	0	Fixed	2.73 (1.40-5.37)	0.003
Laparoscopy	6	0.37	6.6	Fixed	2.17 (1.18-3.96)	0.01
Anastomotic procedure using Stapler	3	0.49	0	Fixed	0.99 (0.57-1.70)	0.97
Gastric conduit Through posterior mediastinal	6	0.51	0	Fixed	0.97 (0.44-2.17)	0.95
Preoperative albumin (mg/dl)	2	0.17	46.8	Fixed	-0.14 (-0.28--0.002)	0.047

Five studies were assessed to be of good quality based on the MINORS, with scores of 19 or more. The other four studies were assessed to be of moderate quality, with scores of 13 or more (**Table 2**).

**Table 2:** Quality assessment by methodological index for non-randomized studies tool.

Author	Stated aim	Inclusion of consecutive patients	Prospective data collection	Endpoint appropriate for study	Unbiased assessment of study endpoint	F/U period approriate for study	Loss to follow-up <5%	Prospective calculation of study size	Adequate control group	Contemporary groups	Baseline aquivalence of groups	Adequate statistical analysis	Total score
Campbell et al. (26)	2	2	0	2	2	2	2	0	2	0	2	2	18
Dalton et al. (21)	2	2	0	2	2	2	2	0	2	0	2	2	18
Karampinis et al. (22)	2	2	0	1	2	2	2	0	2	0	2	2	17
Kitagawa et al. (25)	2	2	0	2	2	2	2	0	2	0	2	2	18
Noma et al. (23)	2	2	0	2	2	2	2	0	2	1	2	2	19
Ohi et al. (24)	2	2	0	2	2	2	2	0	2	1	2	2	19
Guo Jin-cheng et al. (20)	2	2	0	2	2	2	2	0	2	2	2	2	20
Song Xuantong et al. (19)	2	2	2	2	2	2	2	0	2	1	2	2	21
Rao-Jun Luo et al. (18)	2	2	0	2	2	2	2	0	2	1	2	2	19

### Baseline Characteristics

The age, body mean index, history of smoking, pathological type, diabetes mellitus, cardiovascular disease, obstructive lung disease, tumor location, pathological tumor category (T1–2/T3–4), TNM stage (I–II/III–IV), anastomotic procedure using a stapler, and gastric conduit through the posterior mediastinal were comparable in the ICG and control groups. However, the ICG group was lower in ASA I–II status (RR of 0.63, 95% CI 0.4–0.99), neoadjuvant therapy (RR of 0.59, 95% CI 0.41–0.85), and preoperative albumin (WMD −0.14 g/L, 95% CI −0.28–0.002). The ICG group underwent more thoracoscopy (RR of 2.73, 95% CI 1.40–5.37) and laparoscopy (RR of 2.17, 95% CI 1.18–3.96) (**Table 1**).

### Short-Term Clinical Outcomes

The short-term clinical outcomes are summarized in **Table 3**. The operation time and intraoperative blood loss were comparable in the ICG and control groups. There was no significant difference regarding overall postoperative mortality, reoperation rate, arrhythmia rate, vocal cord paralysis rate, pneumonia rate, or surgical wound infection. However, the ICG group had a shorter postoperative stay, with a 2.66-day reduction (WMD, 95% CI −3.77–1.55, p = 0.000).

**Table 3:** Summary of short-term clinical outcomes.

	Included studies	Heterogeneity		Model	Pooled effect	
		*p*	I2%		95% CI	*p*
Intraoperative blood loss (ml)	3	0.41	0	Fixed	-9.18 (-21.34-2.99)	0.14
Operation time (min)	4	0.21	33.1	Fixed	1.69 (-6.86-10.23)	0.7
Postoperative Hospital stay (d)	4	0.44	0	Fixed	-2.66 (-3.77--1.55)	0
AL	9	0.46	0	Fixed	0.29 (0.18-0.47)	0
Surgical Wound infection	2	0.32	0	Fixed	0.66 (0.29-1.49)	0.31
Pneumonia	5	0.51	0	Fixed	0.85 (0.59-1.23)	0.39
Vocalcord paralysis	2	0.27	17.9	Fixed	1.07 (0.98-1.16)	0.13
Arrhythmia	3	0.04	77.5	Random	0.73 (0.47-1.12)	0.15
Reoperation	7	0.70	0	Fixed	0.67 (0.20-2.22)	0.52
Overall mortality	9	0.28	20.5	Fixed	1.23 (0.45-3.39)	0.69

Al, anastomotic leak.

**Figure 2:**
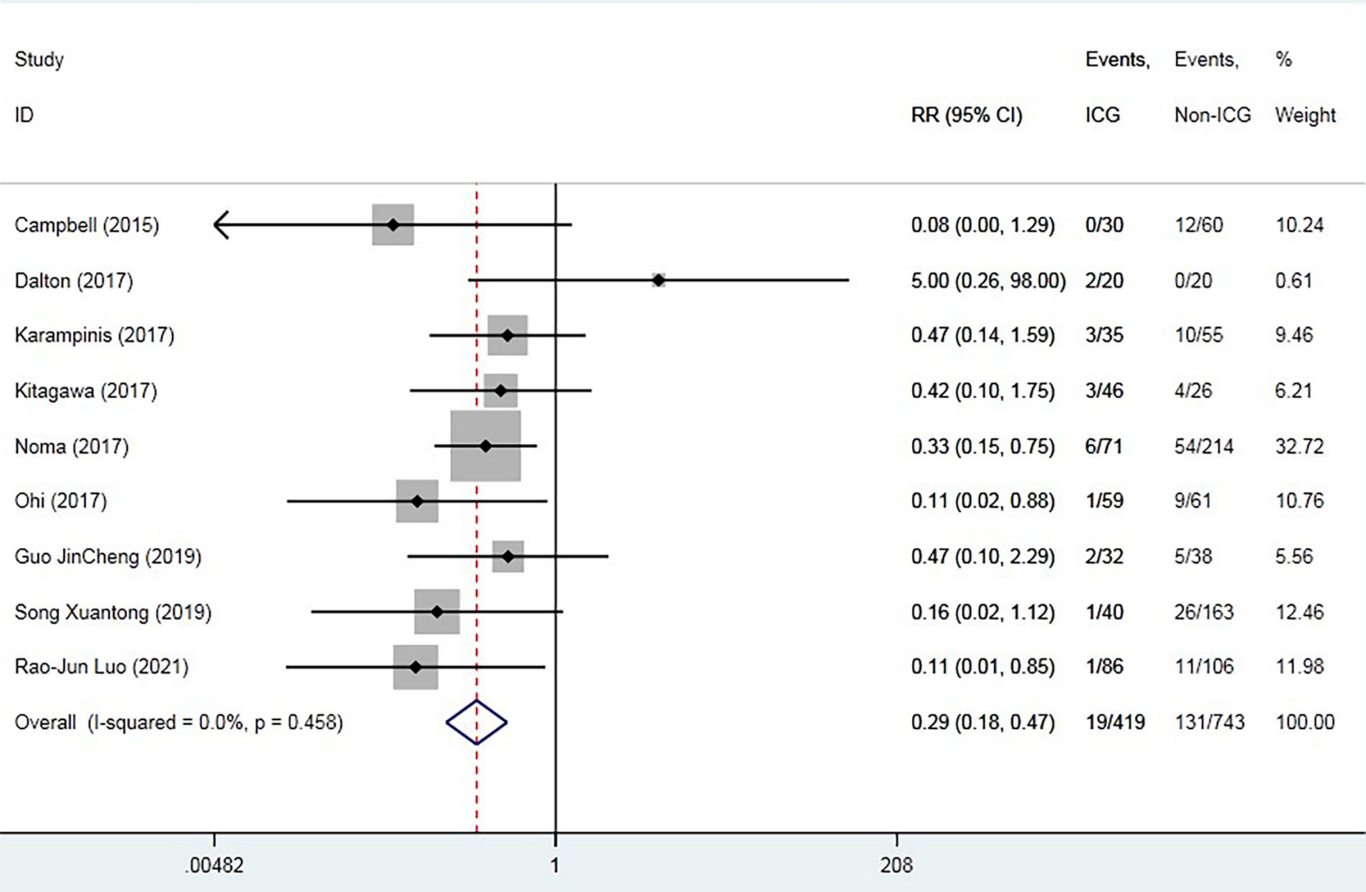
Forest plot for anastomotic leak.

**Figure 3:**
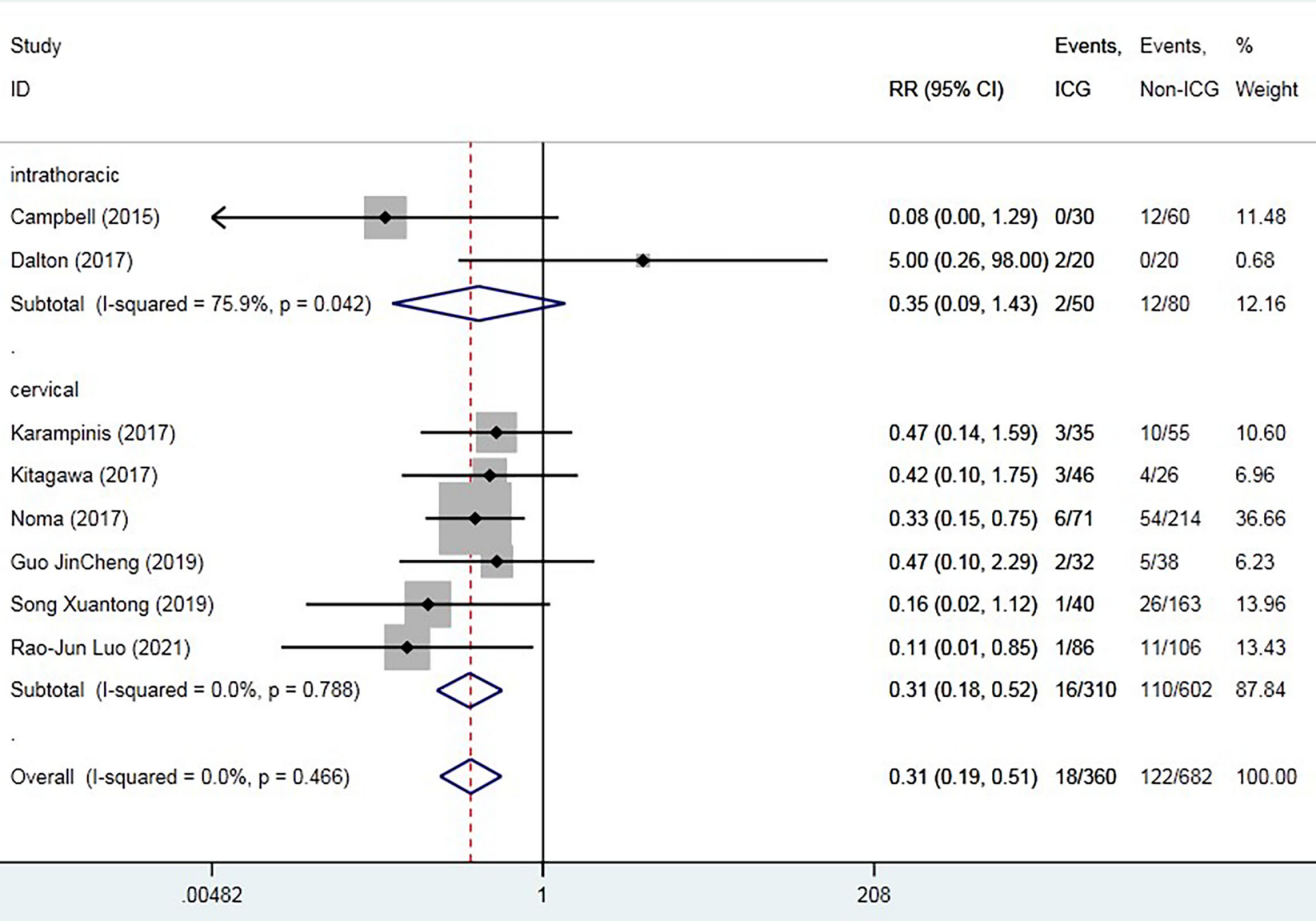
Forest plot for subgroup analysis of anastomotic leak based on anastomosis site.

**Figure 4:**
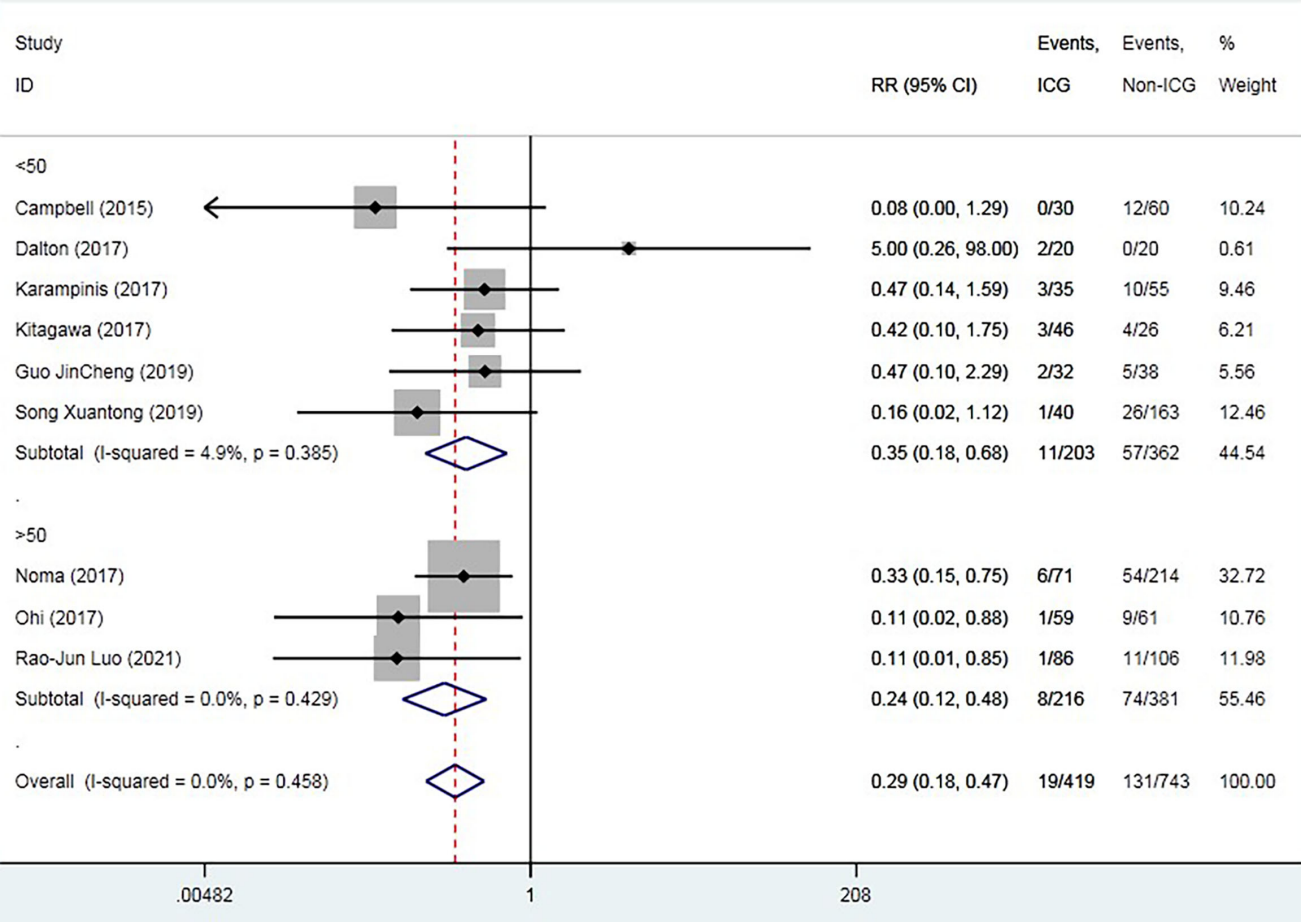
Forest plot for subgroup analysis of anastomotic leak based on experimental group sample size.

**Figure 5:**
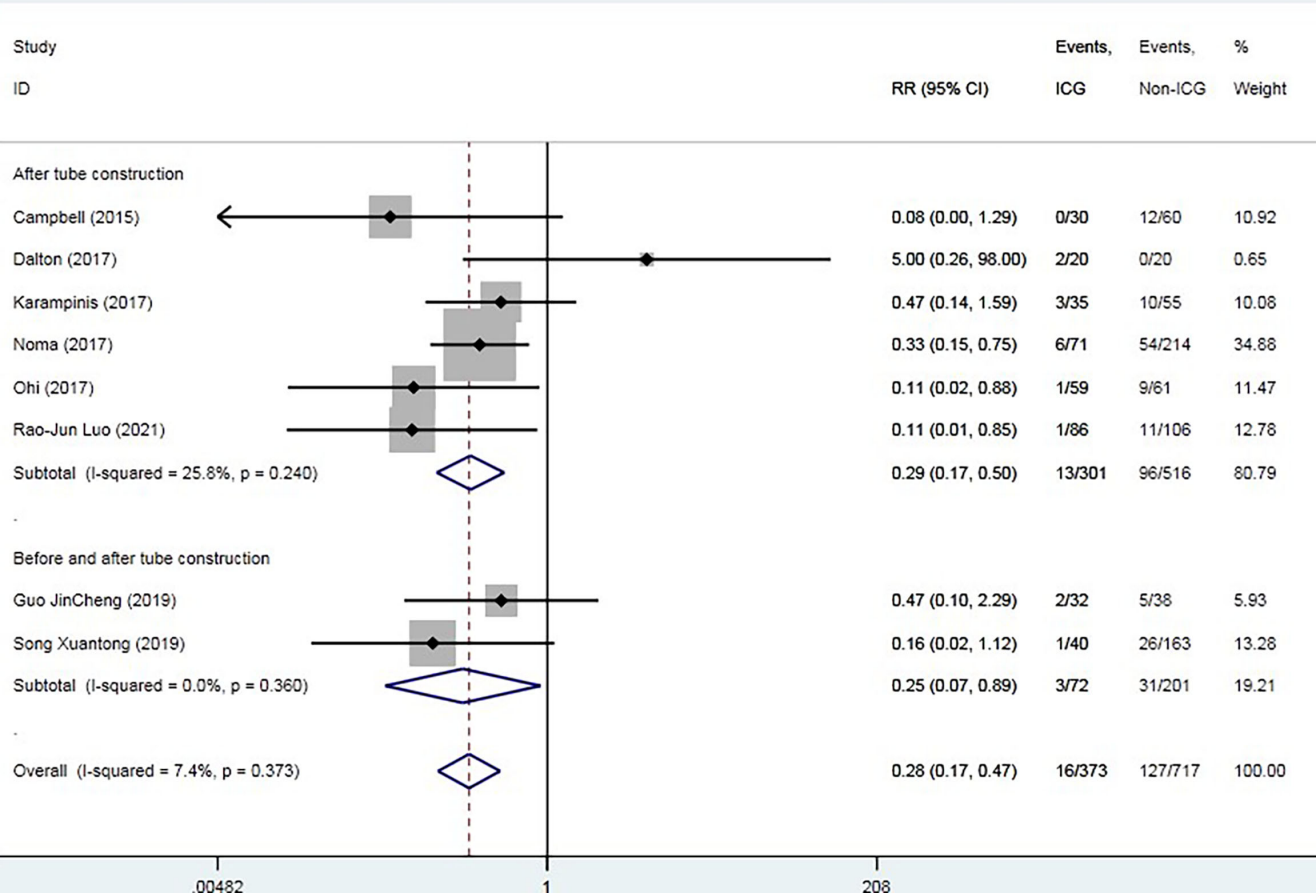
Forest plot for subgroup analysis of anastomotic leak based on indocyanine green fluorescence intervention time.

The overall AL rate was 17.6% (n = 131) in the control group and 4.5% (n = 19) in the ICG group. The overall RR for AL was 0.29 (95% CI 0.18–0.47, p = 0.000), suggesting that ICG was associated with a statistically significant decrease in rates of AL (**Figure 2**). The sensitivity analysis results did not indicate any publications with obvious heterogeneity (**Supplementary 1**). Subgroup analysis was conducted by anastomosis site, ICG group sample size, and intervention time. The application of ICG in cervical anastomosis significantly reduced the incidence of AL (RR of 0.31, 95% CI 0.18–0.52), but the application of ICG fluorescence in intrathoracic anastomosis did not significantly reduce the incidence of AL rate (RR of 0.35, 95% CI 0.09–1.43) (**Figure 3**). Regarding the sample size in the ICG group, publications with a sample size of >50 had a lower RR of 0.24 (95% CI 0.12–0.48). However, publications with a sample size of <50 still had a statistical RR of 0.35 (95% CI 0.18–0.68) (**Figure 4**). Regarding the intervention time of ICG, the application of ICG both before and after gastric construction showed a better RR of 0.25 (95% CI 0.07–0.89) (**Figure 5**).

### Publication Bias

**Figure 6:**
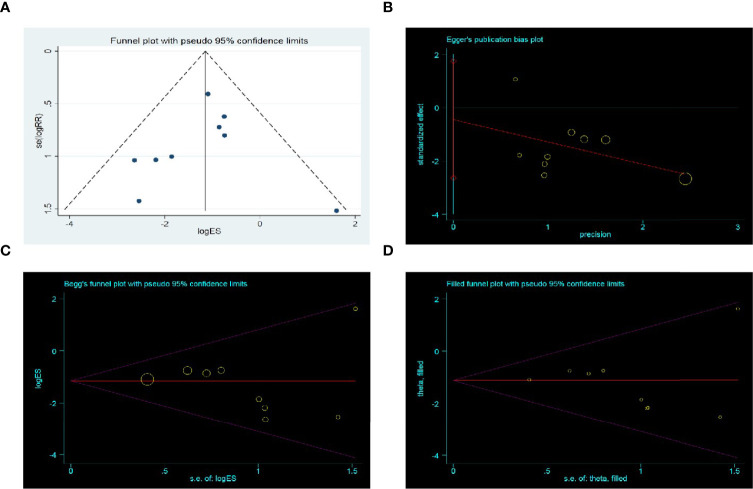
Publication bias assessment. **(A)** Funnel plot; **(B)** Egger’s test funnel plot; **(C)** Begg’s test funnel plot; **(D)** Trim and fill funnel plot. Above four pictures were draw based on anastomotic leak rate.

A funnel plot analysis based on AL was performed. The funnel plot was asymmetrical (**Figure 6A**), which suggested that smaller studies favoring control had been omitted. Thus, we further conducted the Egger’s test (p = 0.65) (**Figure 6B**) and Begg’s test (p = 0.47) (**Figure 6C**), which both indicated no potential publication bias. The trim and fill test was stable without any trim or fill (**Figure 6D**).

## Discussion

This is the first meta-analysis focused on the short-term outcome of ICG fluorescence. No ICG-related adverse events were reported in the included publications. Preliminary study results suggested ICG fluorescence did not increase the operation time or intraoperative blood loss. Overall postoperative mortality, reoperation rate, arrhythmia rate, vocal cord paralysis rate, pneumonia rate, and surgical wound infection were comparable between the ICG and control groups. There was an absolute risk reduction of 69% in the ICG group, which means the prevention of 55 patients in the ICG group from AL. ICG can effectively identify the sufficiency of gastric perfusion so that the surgeon can make an early decision if any adjustment of the gastric tube should be made. This is a promising finding and could explain the reduction in postoperative hospital stays.

The application of ICG could only prevent patients from undergoing cervical anastomosis but not intrathoracic anastomosis from AL (RR of 0.31, 95% CI 0.18–0.52). This contributed to the difference in AL rates between cervical anastomosis and intrathoracic anastomosis. Cervical anastomosis is associated with a significantly increased risk of AL. Biere et al. reported that cervical anastomosis could be associated with a higher leak rate (OR: 3.43; 95% CI: 1.09–10.78; p = 0.03) (27).

There is a consensus that cervical anastomosis requires the formation of a longer gastric tube, which must travel over a longer distance in the mediastinum with a higher tension. This in turn can impair the integrity of the blood vessels around the gastric tube, leading to a greater rate of rupture and leakage (28, 29). For most intrathoracic anastomosis, the blood supply and tension are more likely enough. Thus, using ICG fluorescence to assess blood supply would not be helpful. Another explanation is that the low incidence of AL in intrathoracic anastomosis requires a larger sample size to confirm the validity of ICG.

Although most publications only applied ICG after gastric tube creation, our results showed that the application of ICG both before and after gastric creation showed a better RR of 0.25 (95% CI 0.07–0.89). Kitagawa et al. changed the intervention time from ICG after gastric tube creation to both before and after gastric creation to detect the border of the blood supply (25). Application of ICG before gastric tube creation could help surgeons detect the border of arterial supply and determine the cutting line. In terms of the practical use of ICG in clinical sites, there is no consensus on the dose of ICG. Doses ranged from 1.25 to 25 mg per bolus. The minimum dose can be measured clearly and reliably, but in some cases, it is too low. Higher doses may interfere with the second measurement because the background signal is still high. Slooter et al. recommended a 0.05 mg/kg/bolus dose, and after 15 min, new measurements could be taken (14). However, if we choose to assess gastric perfusion both before and after gastric construction, the total dose of ICG is 25 mg for a patient weighing 50 kg, which is the dose limit of ICG (30). Thus, we recommend 12.5 mg/bolus both before and after gastric construction.

Low volume of the institution and surgeon could be a risk factor of Al (31, 32). Regarding the sample size in ICG group, publications with sample size <50 had a statistical RR 0.35(95% 0.18-0.68), and publications with sample size >50 had a lower RR 0.24(95% 0.12-0.48). This result indicated that application of ICG in gastric blood supply requires a learning curve. Hardy NP et al. conducted a questionnaire about the interpretation of near infrared perfusion imaging using ICG in colorectal surgery in 40 participants, and 70% felt > 10 cases were needed for competency in use with the majority of experts advocating > 50 (33). The learning curve of application of ICG in gastric perfusion is still unclear. Based on this subgroup analysis, application of ICG may have a short learning curve. It seems that, even in learning curve station, ICG application still could prevent patients from AL. Despite the expensive equipment, ICG fluorescence has potential popularization and application value.

### Limitations

Despite the evidence-based findings, there still exist some limitations in our study, Methodological limitations include: (1) Only one study was prospective, and no randomized control trial (RCT) studies were included in meta-analysis. Based on the GRADE scales, the evidence is low. Thus, a well-designed RCT is necessary for further confirm the effect of ICG fluorescence. We would like to update this meta-analysis when there are more reliable studies, especially RCTs. (2) There were some minor differences in baseline data between the ICG group and control group, which may cause potential influence on the incidence of AL. (3) Most studies have not reported AL grading based on the Clavien-Dindo classification. Whether ICG fluorescence could prevent patients from severe AL is unclear. (4) The number of included publications on Ivor-Lewis (conducting intrathoracic anastomosis) is limited, more high-quality publications are necessary to confirm the usefulness of ICG fluorescence in intrathoracic anastomosis. (5) Few studies used ICG quantification to measure the blood flow speed, and most studies ignored the importance of venous congestion. How to reduce the AL rate in patients with poor perfusion is really a question (34). Recently, Takeda FR et.al reported the supercharged cervical anastomosis for esophagectomy procedure may reduce the occurrence of anastomotic leakage and improve perfusion in the anastomotic area via vein and arterial micro-anastomoses based on the ICG quantification technique (35). 

### Conclusion

The application of ICG fluorescence could effectively reduce the incidence of anastomotic leak and shorten postoperative hospital stay for patients undergoing cervical anastomosis, but not for patients undergoing intrathoracic anastomosis. The application of ICG fluorescence before and after gastric management can better prevent patients from AL. This prevention works both in large (>50 cases) and small (<50 cases) medical centers.

## Data Availability Statement

The original contributions presented in the study are included in the article/**Supplementary Material**. Further inquiries can be directed to the corresponding author.

## Author Contributions

All authors listed have made a substantial, direct, and intellectual contribution to the work and approved it for publication.

## Funding

This study was sponsored by Key Laboratory of Cardio-Thoracic Surgery (Fujian Medical University), Fujian Province University, National Natural Science Foundation of China (Grant No. 82070499), National Natural Science Foundation of China (Grant No. 81773129), Joint Funds for the Innovation of Science and Technology, Fujian Province (Grant No. 2020Y9073), Natural Science Foundation, Fujian Province (Grant No. 2020J011036), and Program for Innovative Research Team in Science and technology in Fujian Province University (Grant No. 2018B053).

## Conflict of Interest

The authors declare that the research was conducted in the absence of any commercial or financial relationships that could be construed as a potential conflict of interest.

## Publisher’s Note

All claims expressed in this article are solely those of the authors and do not necessarily represent those of their affiliated organizations, or those of the publisher, the editors and the reviewers. Any product that may be evaluated in this article, or claim that may be made by its manufacturer, is not guaranteed or endorsed by the publisher.
