# Supplementary figures and images for: Indocyanine Green Fluorescence Using in Conduit Reconstruction for Patients With Esophageal Cancer to Improve Short-Term Clinical Outcome: A Meta-Analysis
Source: Front Oncol. 2022 Jun 1;12:847510. doi: 10.3389/fonc.2022.847510 (PMC9198426; doi:10.3389/fonc.2022.847510)

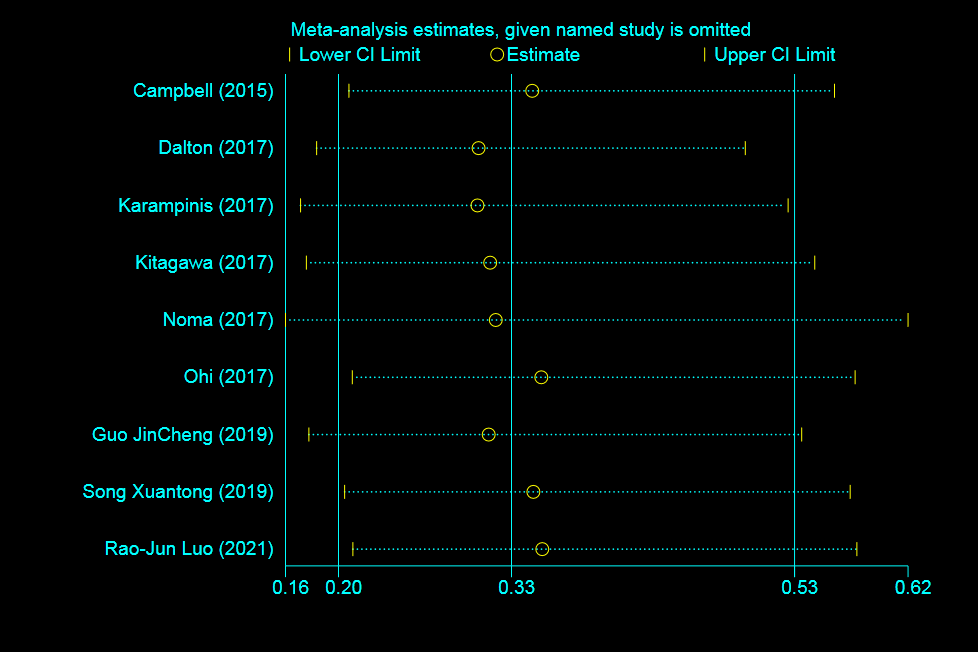


Supplementary 1. Sensitive analysis plot for anastomotic leak.

Supplement: Supplementary file 1 [file DataSheet_1.docx]
